# Reducing Morning MDT Handover Duration Through Structured Communication: A Quality Improvement Project

**DOI:** 10.1192/bjo.2026.11393

**Published:** 2026-06-30

**Authors:** Alex Hodson

**Affiliations:** Oxleas NHS Foundation Trust, London, United Kingdom. South London and Maudsley NHS Foundation Trust, London, United Kingdom

## Abstract

**Aims:**

Effective handover of patient care is essential for patient safety. In psychiatric inpatient wards, morning multidisciplinary team (MDT) handover is used to communicate information from previous shifts; however, this process was criticised locally for lack of structure, prolonged duration and omitted information. This quality improvement project aimed to reduce the duration of morning handover through implementation of a structured handover tool. A secondary aim was to explore the relationship between the number of attendees and handover duration.

**Methods:**

The Model for Improvement methodology was used. Initial qualitative discussions with both junior and senior MDT members identified perceived problems with the existing handover process and informed potential change ideas. Baseline data were collected over approximately 1 month (N=17), recording total handover duration and number of attendees. Comparable data were also collected from four other inpatient wards where structured handovers were already implemented, providing benchmarking.

A structured morning handover tool based on the SBAR framework was developed and implemented, then disseminated to nursing staff responsible for leading the handover. Following implementation, handover duration and attendance were measured over a further one-month period (N=19), with additional data collection (N=5) during two separate weeks in subsequent months to assess sustainability. Qualitative feedback was obtained from the MDT to assess attitudes to the intervention.

**Results:**

Mean handover duration decreased from 68 minutes (range 35–102) to 52 minutes(range 38–78)following implementation, representing a 24% reduction. During months three and four, one-week samples showed mean handover duration of 53 and 48 minutes respectively, demonstrating sustained improvement.

With an average attendance of 9 staff members per handover, this reduction equated to 720 minutes (12 hours) of MDT clinical time released per week. Qualitative feedback was positive, comments included ‘continuous positive influence’, ‘a huge improvement’ and ‘concise without sacrificing thoroughness’.

Baseline handover duration on comparator wards, using alternative structured approaches, ranged from 35–47 minutes, suggesting that using a structured handover rather than the specific format used was the key factor associated with decreased duration, although ward differences likely also contributed.

Correlation coefficients between attendance and handover duration ranged from +0.13 to +0.69 across wards and time points, however no intervention targeting this was implemented.

**Conclusion:**

Implementation of a structured morning handover was associated with a sustained reduction in handover duration and improved perceived quality. The findings suggest that implementing any structured approach is more important than the specific framework used. Increased attendance was associated with longer handover duration.

